# Draft Genome Sequence of Plant Growth–Promoting *Micrococcus luteus* Strain K39 Isolated from *Cyperus conglomeratus* in Saudi Arabia

**DOI:** 10.1128/genomeA.01520-16

**Published:** 2017-01-26

**Authors:** Feras F. Lafi, Juan S. Ramirez-Prado, Intikhab Alam, Vladimir B. Bajic, Heribert Hirt, Maged M. Saad

**Affiliations:** a King Abdullah University of Science and Technology (KAUST), Biological and Environmental Sciences and Engineering Division (BESE), Thuwal, Kingdom of Saudi Arabia; b Computational Bioscience Research Center (CBRC), King Abdullah University of Science and Technology (KAUST), Thuwal, Kingdom of Saudi Arabia

## Abstract

*Micrococcus luteus* strain K39 is an endophyte bacterium isolated from roots of the desert plant *Cyperus conglomeratus* collected from the Red Sea shore, Thuwal, Saudi Arabia. The draft genome sequence of strain K39 revealed a number of enzymes involved in salinity and oxidative stress tolerance or having herbicide-resistance activity.

## GENOME ANNOUNCEMENT

Under the framework of the Darwin21 project (http://www.darwin21.net), extensive microbial isolations from the roots of different desert plants have been conducted and have revealed a number of *Actinobacteria* strains with the potential to promote the growth of *Arabidopsis thaliana* under salt-stress conditions. *Micrococcus luteus* is part of the *Micrococcaceae* family, and its cells are arranged in tetrads. *Micrococcus* spp. have been isolated from soil, air, water, and plant samples (1), and the species *Micrococcus luteus* has been found to have plant growth–promoting properties (2). *M. luteus* strain K39 was isolated from surface-sterilized roots of *Cyperus conglomeratus*, a naturally occurring plant in the desert of the Arabian Peninsula. The plants were collected approximately 10 m from the coast of the Red Sea near Thuwal, Saudi Arabia (22 30 95°N, 39.1047°E). The root extracts were plated on R2A media (3) supplemented with 3% NaCl. Single colonies were subcultured after selection from a 10^−4^ dilution plate grown at 28°C.

Genomic DNA of strain K39 was extracted using a Qiagen DNeasy blood and tissue kit following the manufacturer’s protocol. The DNA was then sequenced by paired-end Illumina MiSeq, and the sequencing library was constructed as described previously (4). Contig assembly was done with SPAdes assembler version 3.6 with a 1-kb contig cutoff size (5). *De novo* assembly of MiSeq reads for *M. luteus* strain K39 resulted in 124 contigs with a total length of 2,513,216 bp and a mean contig size of 20,268 bp. The *N*_50_ was 34,872 bp, and the *L*_50_ was reached with 22 contigs, with an average GC content of 72%. MegaBLAST searches (6) of the K39 strain concatenated genome against the NCBI reference genome database (http://www.ncbi.nlm.nih.gov/genome) revealed that the closest relative genome was *M. luteus* NCTC 2665 (NC_012803.1) with 84% sequence coverage and 98% sequence identity. The annotation of *M. luteus* strain K39 resulted in 1,974 open reading frames (ORFs), four rRNAs, 49 tRNAs, and 24 ncRNAs.

Genome annotation was carried out by the INDIGO pipeline (7) with the exception of ORF prediction made by FragGeneScan (8). Analysis of the genome revealed the presence of multiple enzymes involved in salinity stress and its subsequent oxidative stress. The genome encoded for two enzymes involved in salinity tolerance, namely, asparagine synthase (glutamine-hydrolyzing) (EC: 6.3.5.4) (9) and isochorismate synthase (EC: 5.4.4.2) (10). Other enzymes such as trehalose-phosphatase (EC: 3.1.3.12) (11) and phospholipase D (EC: 3.1.4.4) increased salinity and drought tolerance (12). However, the main trait revealed from this study was the ability of *M. luteus* strain K39 to enhance oxidative tolerance via superoxide dismutase (EC: 1.15.1.1) (13) and ferredoxin NADP reductase (EC: 1.18.1.2) (14, 15). The genome has a number of genes encoding for herbicide-resistance enzymes, such as phosphinothricin acetyltransferase (EC: 2.3.1.183) (16) and acetolactate synthase (EC: 2.2.1.6) (17). Moreover, the K39 genome also encodes for enzymes allowing plants to tolerate glyphosate herbicides, specifically 3-phosphoshikimate 1-carboxyvinyltransferase (EC: 2.5.1.19) (18–22) and protoporphyrinogen oxidase (EC: 1.3.3.4) (23–26).

### Accession number(s).

The genome sequence of *Micrococcus luteus* strain K39 was deposited at DDBJ/EMBL/GenBank under the accession number LWGN00000000. The version described in this paper is the first version, LWGN01000000.
